# Measuring Anxiety in Children: The Importance of Separate Mother and Father Reports

**DOI:** 10.1007/s10566-017-9402-5

**Published:** 2017-04-21

**Authors:** Mélou Jansen, Denise H. M. Bodden, Peter Muris, Marleen van Doorn, Isabela Granic

**Affiliations:** 1 Behavioural Science Institute, Radboud University, P.O. Box 9104, 6500 HE Nijmegen, The Netherlands; 2 Clinical Psychological Science, Faculty of Psychology and Neuroscience, Maastricht University, Maastricht, The Netherlands

**Keywords:** Anxiety disorders, Children, Parent report, Scared, Assessment, Psychometrics

## Abstract

**Background:**

Previous research suggests that it is important to use parental reports when assessing children’s anxiety, but it remains unclear to what extent there are differences between mothers’ and fathers’ scores and whether these potential differences have any repercussions for the psychometric properties of the scale being used.

**Objective:**

This study was conducted to investigate parental differences on the Parent version of the Screen for Child Anxiety Related Emotional Disorders-Revised (SCARED-RP), a rating scale for measuring child anxiety symptoms. The second aim was to re-examine the reliability and validity of the SCARED-RP, in light of these possible differences.

**Methods:**

The SCARED-RP and the Child Behaviour Checklist (CBCL) were administered to parents of clinically anxious children (*n* = 81), and control children (*n* = 108). All children (*n* = 189) completed the SCARED-R.

**Results:**

Significant correlations between mother and father reports were found within the clinically anxious sample. Mothers showed significantly more correspondence with their children in the control group than fathers. The SCARED-RP internal consistency on total scale was excellent (mothers: .94; fathers: .94) and moderate to good for all subscales (from .66 Situational-Environmental Phobia to .93 Animal Phobia). The SCARED-RP differentiated well between clinically anxious and control children (mother and father data). The concurrent validity was supported by strong correlations with the CBCL anxious-depressed scale.

**Conclusion:**

Differences between mother and father reports suggest the importance of obtaining information from both parents separately. Furthermore, the SCARED-RP is a useful instrument for assessing children’s anxiety disorder symptoms in clinical and research settings.

## Introduction

Anxiety is an emotional state characterized by mostly covert symptoms including negative thoughts and increased heart rate. Therefore, it is often difficult to get reliable measures of these underlying anxiety symptoms from outside observers, including parents of potentially anxious children. Instead, it has been argued that children themselves are important informants for the evaluation of their anxiety and its frequency, intensity, and severity. As an initial screening of childhood anxiety disorders, self-report measures are often useful and less time-consuming and costly compared to interviews. One frequently used instrument for assessing child anxiety is the Screen for Child Anxiety Related Emotional Disorders-Revised (SCARED-R; Muris et al. 2007). The SCARED-R is a questionnaire that measures all anxiety disorder symptoms as listed in the DSM classification system and has demonstrated good psychometric properties (e.g., Hale et al. 2011; Muris et al. 2004).

Despite their potential, child self-reports such as the SCARED-R, also have their limitations. First, the level of children’s cognitive and emotional development may have an impact on children’s understanding of the items and the quality of their responses (Breton et al. 1995; Grills and Ollendick 2003). Second, the tendency to provide socially desirable answers may be high, especially for anxious children who often feel embarrassed and uncomfortable when reporting to a clinician about their feelings and thoughts (Silverman and Ollendick 2005). For these reasons, it is recommended to also consult other informants such as parents when assessing anxiety in children.

To assess children’s anxiety symptoms from the parents’ points-of-view, a parent version of the SCARED-R (SCARED-RP) has been constructed (Muris et al. 1999), which has good psychometric properties (Muris et al. 1999, 2004; Hale et al. 2011). Parent reports may contribute information from different perspectives and about different aspects of the disorder, compared to child reports. Although parents are important informants to report on their children’s anxiety, they are often limited in their report of children’s anxiety symptoms as they can only report on the overt manifestations such as the shyness of the child and crying. Furthermore, parent reports might be influenced by multiple factors such as parental anxiety and depression (De Los Reyes 2011). Since both child and parent reports have their limitations, no informant can be designated as the one who reports the “true” number of symptoms (De Los Reyes and Kazdin 2005; De Los Reyes et al. 2015). Relying on only one particular informant rather than integrating information from multiple informants, can lead to different conclusions. Therefore, in clinical practice, information about child anxiety, as provided by children and parents, is often used in a combined, but also haphazard, fashion.

When utilizing this multi-informant approach, clinicians and researchers are often confronted with differences among informant reports and must determine the meaning of such differences and how discrepant reports should be weighted and integrated into one conclusion. Cross-informant correlations for child anxiety symptoms has been a focus of research for decades, where investigators have found relatively low parent child agreement (De Los Reyes and Kazdin 2005; Jensen et al. 1999). A recent study by De Los Reyes et al. (2015) showed a low parent–child agreement on child internalizing problems (*r* = .25). In most cases children tend to report higher levels of anxiety symptoms than their parents (Comer and Kendall 2004; Rapee et al. 1994; Rothen et al. 2009; Russell et al. 2016; Weems et al. 2010). This lack of correspondence is often explained by the proposition that internalizing problems are less salient to parents and thus parents simply are unaware of the true levels of anxiety in their child. The first aim of the current study was to examine differences between parent and child reports of children’s anxiety symptoms with the hypothesis that parents would report fewer symptoms than children themselves.

Within the multi informant literature, a recent debate adds a new emphasis on parent–child disagreement (Russell et al. 2016; Weems et al. 2010). The meaning researchers give to differences comes down to whether these differences are attributed to “real” differences that come from the varied experiences parents and their children have of observing the same phenomena (anxiety) in different contexts versus the inability of children to report on particular feelings like anxiety that are actually there and understood by parents. To illustrate, when filling out a questionnaire, a child reports about their fear in multiple situations and contexts and includes covert manifestations such as their level of anxious feelings during school hours, whereas parents base their perspectives mainly on the children’s behavior at home, such as refusing to sleep at a friend’s house. This implies that observed differences reflects true differences between informants, rather than a measurement error or an inability of the child to understand constructs being measured. This study contributes to this multi-informant discussion by focusing on different perspectives of parent and child of child anxiety. Since children can also report on their feelings in multiple contexts, it is hypothesized that parents underreport their children’s anxiety symptoms compared to their children.

The current study design expanded on previous research in two additional ways. First, a limitation in multi-informant studies is the assumption that both parents share the same perspective. To demonstrate, most studies that include child and parent reports do not distinguish between fathers and mothers (e.g., Bodden et al. 2009; Muris et al. 2004; Dirks et al. 2014; Muris et al. 2004) or use only mother reports (e.g., Manassis et al. 2009; Niditch and Varela 2011; Pereira et al. 2015). Consequently, possible differences between mother and father reports of child anxiety symptoms remain untested. In the current study, we attempted to address these limitations by examining interparental differences on children’s anxiety problems.

There are several reasons why mothers and fathers may see different levels of anxiety in their children. The first reason may be due to differences in the amount of time they spend with their children. It may be that, at least in Dutch households where the majority of mothers work part time, time differences due to work hours means that mothers spend more time with their children than fathers. This gives mothers the opportunity to observe more behavioral problems in their children, compared to fathers, resulting in reporting higher levels of child anxiety (Cinamon and Rich 2002; Treutler and Epkins 2003). Another influencing factor in this discrepancy can be derived from the different, traditional roles both parents play in raising their children, again especially in countries like the Netherlands where mothers are more often likely to take on the majority of parenting practices compared to fathers. For example, mothers are more focused on caring and nurturing, while fathers teach their children more about social competition and risk taking behavior (Moller et al. 2013). Because of these differences in time spent and different roles, it is likely that parents also observe the child and his or her anxiety differently. Studies that have examined differences between fathers and mothers on their reports of their children’s anxiety found that fathers provided a unique perspective on their children’s behavior and emotional problems and rated their offspring as less anxious than mothers (e.g., Dave et al. 2008; Krain and Kendall 2000; Schroeder et al. 2010; Treutler and Epkins 2003). The current study aims to further examine these differences between fathers and mothers. It was hypothesized that fathers would show low overall correspondence with mothers on child anxiety levels and report less anxiety symptoms in their children than mothers.

Finally, the current study aims to contribute to the existing literature on anxiety measurements by reexamining the psychometric properties of the SCARED-RP. Given the possible differences between parents, the need for reliable and valid mother and father reports of anxiety symptoms is high. Furthermore, to find evidence that informant differences represents different perspectives on the same construct, the validity, as well as reliability, was examined. Since it was expected that fathers would report less anxiety, it was hypothesized that the reliability and validity for fathers would also be lower compared to mothers.

## Design and Hypotheses

For the current study, the SCARED-RP was administered to mothers and fathers of clinically anxious children and a control group that combined clinical non-anxious children and non-clinical children. The clinically anxious children were all obtained at clinical agencies. Within the control group, children from clinical agencies without anxiety diagnoses (clinical non-anxious children) were included, as well as children from regular practice without behavioral problems at a clinical level (non-clinical children). The purpose of this study was to investigate (1) the overall correspondence between child- and parent-report and between both parents, and (2) the psychometric properties of the SCARED-RP differentiating between mothers and fathers. The reliability, concurrent and discriminant validity was examined. The specific hypotheses to be considered were:

1. Parents would underreport their children’s anxiety symptoms compared to their children.
2. Fathers would show low overall correspondence with mothers on child anxiety levels and report less anxiety symptoms than mothers.
3. The reliability and validity for fathers would be lower compared to mothers.

## Methods

### Participants

The data in this study originated from a larger study (Jansen et al. 2012) which investigated the effectiveness of an individual CBT program called *Thinking* + *Doing* = *Daring* (*Denken* + *Doen* = *Durven*; Bögels 2008). In the current study, three groups were initially planned to be investigated: Participants in the clinically anxious group (1) were 81 mothers and 55 (68%) fathers of clinically referred children with an anxiety disorder. This group consisted of 40% boys (n = 32) and 60% girls (n = 49) and had an average age of 10.4 years (SD = 1.36). The majority of boys had an Anxiety Disorder-Not Otherwise Specified (56%) or a Generalized Anxiety Disorder (25%). One third also had comorbid Attention Deficit Hyperactivity Disorder (ADHD). Among girls, the most frequent diagnoses were Anxiety Disorders-Not Otherwise Specified (39%), Social Phobia (25%), and Generalized Anxiety Disorder (22%). Only 18% of the clinically anxious girls had a comorbid disorder besides anxiety, mostly Depressive and/or Dysthymic Disorder (see Table 1).

**Table 1:** Percentage anxiety and comorbid DSM-diagnoses in the clinically anxious (*n* = 81) and (clinical non-anxious) control (*n* = 31) sample

	Clinically anxious				Clinical non-anxious control			
	Boys		Girls		Boys		Girls	
	*n*	%	*n*	%	*n*	%	*n*	%
Social Phobia	3	9.5	12	25				
Separation anxiety disorder	3	9.5	5	10				
Generalized anxiety disorder	8	25	11	22				
Panic disorder	0	0	1	2				
Specific phobia	0	0	1	2				
Anxiety disorder NOS	18	56	19	39				
No comorbid diagnosis	13	54	17	82				
Attention-deficit hyperactivity disorder	7	29	1	2	16	70	3	37.5
Oppositional defiant disorder	1	4	1	2	1	4	0	0
Conduct disorder	0	0	0	0	1	4	2	25
Depressive and/or dysthymic disorder	0	0	2	6	0	0	0	0
Pervasive development disorder	0	0	0	0	3	13	0	0
Other comorbid diagnosis	3	13	3	8	2	9	3	37.5

The clinical non-anxious control group (2) consisted of 31 mothers and 25 (81%) fathers and their clinically referred (but not anxious) children. This group included 74% boys (n = 32) and 26% girls (n = 8) with an average age of 10.2 years (*SD* = 1.52). Most boys and girls had ADHD (resp. 70 and 37.5%).

Participants in the non-clinical control group (3) were 77 mothers and 61 (79%) fathers of non-referred children without a disorder. The non-clinical control group contained 40% boys (n = 31) and 60% girls (n = 46) who had an average age of 10.3 years (*SD* = 1.79). All participants were Dutch residents. Most children were Caucasian and from middle class families. Unfortunately, due to recruitment problems inherent in “real world” clinical settings, we did not collect data from enough clinical non-anxious families to conduct the intended analyses with enough power. Therefore, we collapsed the two control groups into one for all of the analyses.

### Procedure

The clinical sample (anxious and non-anxious) was obtained by including children who were referred to a child mental health center. These children, as well as their parents, were asked to participate and to fill out an informed consent form, and explained that there was no obligation to participate. Next, consenting families were asked to fill out questionnaires, as an added part of the routine diagnostic process used in each child mental health center. All parents who filled out the questionnaires were instructed to fill out the questionnaires separately. The children (*N* = 189) filled out the questionnaires in the same week as their parents and, if applicable, before treatment. Only children with an anxiety disorder were included in the clinically anxious sample.

To obtain a sample from the general population, parents and their non-clinical children were invited to participate by Radboud University graduate students. Only children with a non-clinical back ground, not diagnosed with an anxiety disorder and not in current treatment, were included. Again after completing the consent form, child and parents filled out questionnaires.

Ethical approval has been granted by the ethical committee of the Faculty of Social Sciences at the Radboud University Nijmegen (ECG16122010). This study was also registered in the Dutch Trial Register: NTR2967 and conformed to CONSORT guidelines.

### Measures

#### SCARED-RP

The SCARED-RP is a parent version of the SCARED-R and screens for child anxiety symptoms by means of 69 items (e.g., “My child is afraid of the dark”) (Bodden et al. 2009). Seven subscales are included in the SCARED-RP: Separation Anxiety Disorder, Panic Disorder, Specific Phobia, Social Phobia, Obsessive Compulsive Disorder, Post Traumatic and Acute Stress Disorder and Generalized Anxiety Disorder. Parents score each symptom on a three-point scale: hardly ever (0), sometimes (1), or often (2). The internal consistency has been shown to be satisfactory, in clinical as well as non-clinical populations (α > .70; e.g., Hale et al. 2011, 2005). Also support for its’ discriminant validity (clinically anxious children reported significantly more anxiety symptoms than control children; Bodden et al. 2009) and concurrent validity was found (children with anxiety disorders displayed higher levels of anxiety disorders symptoms than children with other problems; Muris et al. 2004).

#### SCARED-R

The SCARED-R (child version) contains the same 69 items, but assesses anxiety from the children’s perspective (e.g., “I am afraid of the dark”). Both SCARED-R and SCARED-RP Total score and subscale scores can be obtained by summing across relevant items. Previous studies show that the SCARED-R is reliable and valid (Muris et al. 2004; Muris and Steerneman 2001). The internal consistency (α > .72; Muris et al. 2004) of the scale was satisfactory, the concurrent validity was supported (SCARED-R total scores were significantly associated with CBCL-internalizing problems (rs between .26 and .58, all Ps < .05), but not with CBCL-externalizing problems (rs between .11 and .10 in Muris et al. 2004), and there was also evidence for its discriminant validity (children with anxiety disorders displayed higher levels of anxiety disorders symptoms than children with other problems; Muris et al. 2004).

#### CBCL

Besides the SCARED-RP, parents completed the Child Behavior Checklist (CBCL; Achenbach and Ruffle 2000). This questionnaire assesses internalizing and externalizing behavior problems of children. The CBCL consists of 113 items. Both mother and father were asked to rate each item on a 3-point scale ranging from 0 (does not apply to the child) to 2 (clearly or often). The CBCL showed satisfactory psychometric properties (Achenbach and Ruffle 2000; De Groot et al. 1994). In our study, strong internal consistencies were found for the CBCL subscales. Cronbach’s alphas on the subscales were between .76 and .88 for mother report and between .71 and.84 for father report. Only one relatively weak internal consistency was found (α = .62) on mother report on the subscale Somatic problems.

#### DSM-Diagnoses

Clinicians of all clinical children were asked to report the DSM-diagnosis based on an extensive diagnostic process. Each of the clinical agencies laid out a standardized diagnostic procedure which was followed by all participating clinicians. All clinicians used one or more interview sessions with the parents and with the child to construct a detailed description of all problem behavior occurring in several surroundings. Additionally, questionnaires like the Child Behavior Check List and Teacher Report Form were filled out. When required, a home or school observation was carried out. After combining all of the information provided, a temporary DSM-diagnoses was given. This diagnosis was then discussed by colleagues, before setting the final diagnosis.

### Statistical Analysis

First, missing data on the item level were imputed using the missing value analysis with regression in SPSS. No more than 8% of the items were missing per questionnaire. T-tests were performed to determine whether fathers that participated (*n* = 141) differed from fathers that refused, or were not able to participate (*n* = 48). These groups did not differ on child age or gender or mother-total SCARED score, so all further analyses were executed with all participants. Both control groups (clinical non-anxious and non-clinical) were merged into one control group for all analyses, to maximize power. One-way analysis of variance (ANOVA) were performed to test for gender differences. In the clinically anxious sample, both parents reported girls higher on panic symptoms than boys. In the control group both parents reported girls higher on Situational-Environmental Phobia than boys, whereas only fathers reported girls to exhibit higher levels of animal phobia as compared to boys. Although these differences were relatively minor compared to the number of comparisons, gender was entered as a covariate for all subsequent analyses. ANOVA’s were also performed to test for age differences, but no differences were found. Accordingly, age was not used as a covariate.

Second, to test the overall correspondence between child- and parent-report and between both parents, partial correlations were computed to test for cross-informant correspondence. To assess the significance of the difference between the two correlation coefficients, z-values were calculated. One-way analysis of (co)variance (ANCOVA) was used to test for child differences in parent reports and to compare various groups on the SCARED total scale.

Third, the following analyses were carried out to test the psychometric properties of the SCARED-RP. To determine internal consistency of the total score as well as on all subscales, Cronbach’s alphas were computed. To examine the concurrent validity, SCARED-RP Total scores were correlated with the CBCL DSM-oriented subscale Anxiety problems. Discriminant validity was first tested by one-way ANCOVA to compare parental reports between groups. To evaluate differences on the subscales, a MANCOVA was carried out. An additional test for discriminant validity was conducted by calculating correlations between the SCARED-RP Total scale and CBCL DSM-oriented subscales.

## Results

### Overall Correspondence Between Mother–Father and Parent–Child

As shown in Table 2, significant correlations between mother and father reports were found on the Total score and all subscales within the clinically anxious sample. In the control group, only one non-significant correlation was found (Panic Disorder). On two subscales correlations between the clinically anxious sample and control sample were significantly different. Parents showed significantly more correspondence on Panic Disorder and Situational-Environmental Phobia in the clinically anxious sample compared with the control sample.

**Table 2:** Partial correlations and Z-scores, controlled for gender, of SCARED-P scores between informants in the clinically anxious and control sample

	Mother–father correspondence			Parent–child correspondence					
	Clinically anxious	Control	Z	Clinically anxious		Z	Control		*Z*
				Mothers	Fathers		Mothers	Fathers	
	n = 55	n = 86		n = 81	n = 55		n = 108	n = 86	
	*r*	*r*		*r*	*r*		*r*	*r*	
Total score	.61**	.52**	.75	.55**	.46**	.68	.53**	.24*	2.35*
Subscales									
Generalized anxiety disorder	.67**	.46**	1.77	.49**	.47**	.15	.52**	.22*	2.4*
Separation anxiety disorder	.64**	.64**	0	.59**	.52**	.57	.49**	.38**	.93
Social Phobia	.49**	.67**	−1.55	.55**	.53**	.16	.66**	.40**	2.51*
Panic disorder	.64**	.06	3.95**	.55**	.42**	.95	.11	-.01	.82
Obsessive compulsive disorder	.66**	.49**	1.45	.60**	.48**	.95	.53**	.19	2.71**
Post traumatic stress disorder	.44**	.44**	0	.30*	.08	1.28	.33**	.21	.88
Animal phobia	.69**	.68**	.11	.58**	.53**	.4	.38**	.34**	.31
Medical phobia	.69**	.51**	1.61	.47**	.47**	0	.34**	.25*	.67
Situational-environmental phobia	.79**	.43**	3.46**	.67**	.61**	.57	.51**	.22*	2.31*

** *p* < .01; * *p* < .05

In the clinically anxious sample, high correspondence between parents and children was found on most of the subscales, except for Post-Traumatic Stress Disorder. Only mothers agreed to some extent with children on that scale. However, the correlations between both parents didn’t differ significantly. In the control sample, mothers corresponded more with their children than fathers. On the Total scale and all subscales, mothers corresponded moderate to high with their children. No correlations were found between fathers and their children on Panic Disorder, Obsessive Compulsive Disorder and Post Traumatic Stress Disorder. Mothers showed significantly more correspondence with their children in the control group than fathers on the total scale, as well as on four subscales (e.g., Generalized Anxiety Disorder and Social Phobia).

### Psychometric Properties of the SCARED-RP

#### Internal Consistency

In the total sample, the internal consistencies for the full scale were excellent (mothers: .94; fathers: .94; see Table 3) and moderate to good for all subscales. The lowest internal consistency was found for the Situational Environmental Phobia subscale (mothers: .70; fathers: .66) and Obsessive Compulsive Disorder subscale (fathers: .68). When examining reliability within separate groups, moderate to good internal consistencies were found for both parents of clinically anxious children. In general, moderate to good reliability was also found within the control group. Only on some subscales, weak internal consistencies were found within the control group (e.g., .50 for Panic disorder with mother reports, and .59 for the Situational Environmental Phobia with father reports). It can be concluded that the SCARED-RP showed satisfactory reliability on the total score and on most subscales.

**Table 3:** Cronbach’s alpha values for the SCARED-P total and subscale scores for the mother and father for the total sample and two subgroups

	Total sample		Clinically anxious group		Control group	
	Mother	Father	Mother	Father	Mother	Father
	n = 189	n = 141	n = 81	n = 55	n = 108	n = 86
	*α*	*α*	*α*	*α*	α	α
Total score	.94	.94	.90	.92	.93	.93
Subscales						
Generalized anxiety disorder	.88	.86	.83	.87	.84	.81
Separation anxiety disorder	.79	.79	.65	.75	.77	.73
Social Phobia	.87	.86	.82	.83	.86	.85
Panic disorder	.80	.80	.77	.80	.50	.74
Obsessive compulsive disorder	.71	.68	.63	.60	.59	.71
Post traumatic stress disorder	.82	.78	.84	.75	.73	.80
Animal phobia	.89	.93	.91	.95	.83	.91
Medical phobia	.74	.72	.77	.76	.66	.64
Situational-environmental phobia	.70	.66	.67	.69	.66	.59

#### Concurrent Validity

Strong concurrent validity was found for both mother and father reports, in all groups (see Table 4). In the clinically anxious sample, as well as in the control group, mothers’ and fathers’ SCARED scores correlated high with the CBCL DSM-oriented subscale Anxiety problems.

**Table 4:** Partial correlations, controlled for gender, of SCARED-RP total scores between CBCL DSM-oriented subscales in the clinically anxious sample and the control sample

	Mothers		Fathers	
	Clinically anxious	Control	Clinically anxious	Control
	n = 81	n = 108	n = 55	n = 86
	*r*	*r*	*r*	*r*
Anxiety problems	.40**	.62**	.38**	.48**
Affective problems	.24*	.54**	.30**	.29**
Somatic problems	.07	.28**	.32**	.20
Attention deficit/hyperactivity problems	.20	.27**	.22	.16
Oppositional defiant problems	.24*	.31**	.15	.24*
Conduct problems	.03	.33**	.11	.20

* *p* < .05; ** *p* < .01

#### Discriminant Validity

Results showed that mothers of clinically anxious children reported significantly more anxiety symptoms on the Total score and on all subscales compared to mothers of control children (see Table 5). Fathers of clinically anxious and control children reported significantly more anxiety symptoms on the Total score. Furthermore, significant differences on all subscales between these groups with father report were found, with the exception of Post Traumatic Stress Disorder and Animal Phobia.

**Table 5:** Mean SCARED-RP scores and standard deviations in the clinically anxious and the control group with comparison between groups, controlled for gender

Mother version	Clinically anxious		Control		*F*-*value*	*η*_*p*_^*2*^
	n = 81		n = 108			
	*M*	*SD*	*M*	*SD*		
Total score	42.1	16.5	18.2	13.3	117.0**	.39
Generalized anxiety disorder	8.1	4.0	3.5	3.1	75.5**	.29
Separation anxiety disorder	8.0	4.3	3.1	3.2	76.0**	.29
Social Phobia	6.5	3.3	3.3	2.8	48.8**	.21
Panic disorder	4.4	3.7	1.1	1.3	73.6**	.28
Obsessive compulsive disorder	3.6	2.7	1.0	1.5	69.5**	.27
Post traumatic stress disorder	2.1	2.1	.9	1.4	21.7**	.10
Animal phobia	1.3	1.9	.6	1.2	9.6**	.05
Medical phobia	3.9	3.2	2.4	2.3	13.4**	.07
Situational-environmental phobia	3.2	2.5	1.7	1.8	21.8**	.10

Father version	n = 55		n = 86		*F*-*value*	*η*_*p*_^*2*^
	*M*	*SD*	*M*	*SD*		
Total score	29.9	16.7	16.1	13.0	27.8**	.17
Generalized anxiety disorder	5.6	4.2	3.1	2.9	15.5**	.10
Separation anxiety disorder	5.8	4.2	2.6	2.7	28.9**	.17
Social Phobia	5.1	3.4	2.8	2.5	18.1**	.12
Panic disorder	2.9	3.3	1.2	1.9	13.7**	.09
Obsessive compulsive disorder	2.5	2.3	1.0	1.7	21.1**	.13
Post traumatic stress disorder	1.2	1.6	.7	1.3	3.6	.03
Animal phobia	.8	1.7	.7	1.3	.1	.00
Medical phobia	2.8	2.9	1.8	2.0	5.2*	.04
Situational-environmental phobia	2.4	2.3	1.5	1.6	5.1*	.04

** *p* < .01; * *p* < .05

Another test of discriminant validity was carried out by calculating correlations between the SCARED-RP Total scale and the CBCL DSM-oriented subscales Affective problems, Somatic problems, Attention Deficit/Hyperactivity problems, Oppositional Defiant problems and Conduct problems (see Table 4). In the clinically anxious group, most correlations were non-significant, indicating good discriminant validity. However, between mother reported total anxiety symptoms and CBCL DSM-oriented subscales Affective problems and Oppositional Defiant problems significant correlations were found. When using father total anxiety scores, most correlations were also non-significant except between father reported total anxiety symptoms and CBCL DSM-oriented subscales Affective problems and Somatic problems, which were moderate. In the control group, all correlations were significant between mother reported anxiety symptoms and the CBCL DSM-oriented subscales. On father reported anxiety symptoms, no significant correlations were found between the SCARED-RP Total scale and three CBCL DSM-oriented subscales (e.g., Somatic problems).

## Discussion

The present study was designed to examine overall correspondence between father and mother reports on child-anxiety and the psychometric properties of the parent version of the Screen for Child Anxiety Related Emotional Disorders (SCARED-RP), differentiating between mothers and fathers. Unexpectedly, results showed strong mother–father overall correspondence and reasonable parent–child correspondence between mother and child. Next, as predicted, the SCARED-RP was shown to have satisfactory psychometric properties for both father and mother reports.

Our first finding of interest was the cross-informant correspondence. Our findings showed that mothers corresponded more with child reports than fathers in both samples. Similarly, Seiffge-Krenke and Kollmar (1998) found that mothers agreed more with their children on reporting internalizing and externalizing symptoms than fathers. In general, strong overall correspondence between mother and father reports was found in the clinically anxious sample, while the cross-informant correspondence in the control sample showed more variance. It is likely that when anxiety levels reach clinical significance, and in the context of visiting a mental health agency, parents are likely to have spoken about and agreed to the extent of help their child needs for anxiety symptoms. In non-clinical contexts, more variability may be expected given the lack of identified problem with the child. In line with this reasoning, Krain and Kendall (2000) found that parents were likely to report their children as anxious when they are seeking help for those problems. Note also that moderate to high correspondence between parents and their clinically anxious children was found in this study, which is in line with earlier research (Dirks et al. 2014; Muris et al. 2004). Unlike recent studies on cross-informant correspondence, where they used tests of measurement invariance (e.g., Dirks et al. 2014), we used correlations to report on correspondence between parents and children. Low correlations between child and parent reports, in this case father reports, do not automatically lead to the conclusion that the SCARED-RP is measuring fundamentally different constructs if the measurement validity is strong. It must be noted that validity, among other psychometric properties, of the SCARED-RP was also tested and discussed below.

Our second aim was to examine the psychometric properties of the SCARED-RP. As to the reliability of the SCARED-RP, internal consistency was found to be satisfactory on both the Total scale as well as on most subscales in the clinical sample. In line with previous research (Bodden et al. 2009), relatively low internal consistency coefficients were found for the Situational Environmental Phobia subscale. This could be explained by the fact that this subscale included a heterogeneous set of stimuli. For fathers, the reliability of the subscale Obsessive Compulsive Disorder was weak. This scale measured both obsessions and compulsions. Obsessions are hard to observe and might only manifest in subtle behavioral changes and may only be noticed through long, consecutive hours of observations, consistent in this case with Dutch mothers who are by far the more likely of the parents to spend more time with the child (Cinamon and Rich 2002; Treutler and Epkins 2003). In general, it seems fair to conclude that the SCARED-RP is a reliable instrument for measuring child anxiety symptoms from the perspectives of both the father and the mother (see also Birmaher et al. 1999; Bodden et al. 2009).

To establish the concurrent validity of the SCARED-RP, its total score was correlated with the CBCL DSM-oriented subscale Anxiety problems. Good concurrent validity was found, replicating previous studies (Monga et al. 2000; Muris et al. 2004). This provided evidence that observed cross-informant differences were not measurement errors but represented different perspectives on the same construct, which contributes to the understanding of multi-informant differences.

Concerning the discriminant validity of the SCARED-RP, both mother and father reports differentiated well between clinically anxious children and control children on the Total scale. This highlighted the specificity of the SCARED-RP; it measured symptomatology specific to anxiety disorders and not to more general mental health problems. As expected, more child anxiety symptoms were reported by parents in the clinically anxious group as compared to parents in the control group, which is in line with earlier research (Muris et al. 2004; Nauta et al. 2004). The discriminant validity of the SCARED-RP was particularly good for mother reports, which were also capable of discriminating clinically anxious and control children at subscale levels, in contrast to father reports, which differentiated less between the two groups. Among the speculations we might be able to make to explain these results, it is possible that children tend to seek the comfort of their mothers over their fathers when seeking help for an emotional problem or to discuss fears and worries (Almeida and Galambos 1991). Indeed, Youniss and Smollar (1985) found that children identified their mothers as the one person knowing them best, perhaps leading to mothers’ more sensitive abilities to differentiate levels of anxiety.

Furthermore, discriminant validity was additionally tested by comparing the SCARED-RP Total scale and the CBCL DSM-oriented subscales. The SCARED-RP Total scale was not associated with most of the CBCL DSM-oriented subscales, which indicated the generally reasonable discriminant validity for the SCARED-RP. Two comparisons showed lower discriminant validity, however. First, in the clinically anxious group, we found significant correlations between the SCARED-RP Total scale and the CBCL DSM-oriented subscale Affective problems, using both parent reports. Research indicates, that there is a high comorbidity and a substantial overlap between anxiety and depressive symptoms (Seligman and Ollendick 1998). Anderson and Hope (2008) reviewed evidence for a tripartite model, first proposed by Clark and Watson (1991), which describes the underlying similarity between anxiety and depression namely both share a component of negative affect. This overlap may account for the poor discriminant validity found in this study.

Second, in the clinically anxious sample, poor discriminant validity was also found when comparing the maternal reported SCARED-RP Total scale and the CBCL DSM-oriented subscale Oppositional Defiant problems. Mothers reported their child as both high anxious and high oppositional. There are some indications that there is a moderate comorbidity between anxiety disorders and oppositional deviant disorders (ODD) (Chavira et al. 2008), which can account for the co-occurrence of these disorders. Research has shown (Barrett et al. 1996) that children diagnosed with ODD were just as likely to interpret threat in an ambiguous situation as children diagnosed with an anxiety disorder, indicating a shared mechanism between these disorders (for a review, see Granic 2014). Furthermore, social withdrawn children can react aggressively in stressful interactions (Burgess et al. 2006), underlining the possible comorbidity between anxiety and oppositional behavior.

## Limitations and Future Directions

There are a number of limitations of this study that should be acknowledged. First, children’s DSM anxiety diagnoses were provided by clinicians after an extensive diagnostic process that was used in usual clinical care, but they were not based on a structured diagnostic interview. Future studies should use structured diagnostic interviews to be more confident about the accuracy and reliability of our findings (Rettew et al. 2009). Secondly, the number of participants in the study was relatively low, which decreased our statistical power and thereby limits the generalizability of our findings. Third, we did not correct for the amount of time spent with the child. Therefore, the differences found between parents could possibly be biased by the amount of time spent with the child rather than any particular properties of mother vs father relationships. Last, possible gender differences and differences in cut-off scores were not explored in this study due to limited samples sizes. In a larger sample, these differences might emerge and show interesting patterns of relations with different informants.

## Conclusion

The results of this study are encouraging for clinicians and researchers who intend to use the SCARED-RP for measuring children’s anxiety symptoms in mental health care as well as for research purposes. This study showed that the SCARED-RP was a reliable and valid instrument for both mother and father separately. Differences were found between mother and father reports, highlighting the recommendation to obtain information from both parents separately. Relying on only one informant can result in different conclusions. Although, in this study mothers tended to have more overall correspondence with their children, each informant can still be considered as providing unique and important data. These data can subsequently be used for intervention purposes that may include educating the parent for whom their child’s anxiety is not as evident, initiating a discussion between parents to examine potential discrepant perceptions, and helping the child express his or her emotional distress to the parent that may be less aware of these issues (if that is clinically warranted).
